# *Rickettsia japonica* Infections in Humans, Xinyang, China, 2014–2017

**DOI:** 10.3201/eid2509.171421

**Published:** 2019-09

**Authors:** Hao Li, Pan-He Zhang, Juan Du, Zhen-Dong Yang, Ning Cui, Bo Xing, Xiao-Ai Zhang, Wei Liu

**Affiliations:** Beijing Institute of Microbiology and Epidemiology, Beijing, China (H. Li, P.-H. Zhang, B. Xing, X.-A. Zhang, W. Liu); Peking University, Beijing (J. Du);; People's Liberation Army 990 Hospital, Xinyang, China (Z.-D. Yang, N. Cui)

**Keywords:** spotted fever group rickettsioses, emerging infectious diseases, ticks, China, *Rickettsia japonica*, bacteria, signs and symptoms, laboratory tests, thrombocytopenia, hepatic aminotransferases, vector-borne infections, tickborne disease

## Abstract

During 2014–2017, we screened for *Rickettsia japonica* infection in Xinyang, China, and identified 20 cases. The major clinical manifestations of monoinfection were fever, asthenia, myalgia, rash, and anorexia; laboratory findings included thrombocytopenia and elevated hepatic aminotransferase concentrations. Physicians in China should consider *R. japonica* infection in at-risk patients.

Emerging and reemerging spotted fever group (SFG) rickettsioses are increasingly spreading and being recognized worldwide. Japanese spotted fever (JSF), caused by *Rickettsia japonica* and first reported in Japan in 1984 (*1*,*2*), is an SFG rickettsiosis characterized by fever, malaise, chills, headache, rash, and eschars (*3*,*4*). JSF cases have been increasingly reported, but mostly in Japan and the nearby countries of South Korea, the Philippines, and Thailand (*4*–*7*). *R. japonica* has been detected in 3 genera (*Haemaphysalis*, *Dermacentor*, *Ixodes*) and 8 species of ticks (*8*,*9*). Because of the wide spectrum of ticks in which this bacterium has been found, *R. japonica* is postulated to be distributed more widely throughout China. The identification of *R. japonica* in *H*. *flava* and *H*. *hystrici*s ticks in Wuhan, China, further corroborated this hypothesis (*10*). Also, serologic responses to *R. japonica* were detected in patients in Hainan Province in southern China ≈3 decades ago (*11*). However, laboratory-confirmed JSF cases have been identified in only a few patients in China (*12*,*13*). In this study, we aimed to determine if more JSF cases were occurring in China by using hospital-based surveillance.

## The Study

During March 2014–June 2017, we screened 2,236 febrile patients seeking treatment at The People’s Liberation Army 990 Hospital in Xinyang, Henan Province, eastern central China (Appendix Figure) with a history of tick bite or field activity within the previous month for infection with *R. japonica*. We extracted DNA from peripheral blood samples collected during the acute phase of illness and screened them by a nested PCR concurrently targeting the citrate synthase gene (*glt*A), 17-kDa antigen–encoding gene (*htr*A), and outer membrane protein A–encoding gene (*omp*A), as previously described (*14*). To minimize the risk for contamination, we performed template isolation and PCR setup in separate rooms with specified pipette sets, used certified DNAase- and RNase-free filter barrier tips to prevent aerosol contamination, and performed all PCR assays with appropriate controls. After amplification and sequencing, we found that 20 patients were infected with *R. japonica*.

**Figure 1:**
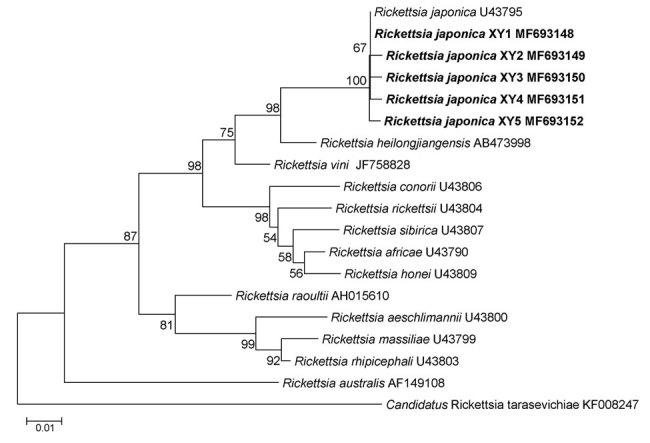
Phylogenetic analysis of *Rickettsia* species from febrile patients treated at The People’s Liberation Army 990 Hospital for *Rickettsia japonica* infection, Xinyang, China, March 2014–June 2017 (bold), and reference species. Tree was constructed on the basis of the outer member protein A nucleotide (311-bp) gene sequence. We used the maximum-likelihood method with the best substitution model (Tamura 3-parameter plus gamma) and MEGA version 5.0 (http://www.megasoftware.net). We applied a bootstrap analysis of 1,000 replicates to assess the reliability of the reconstructed phylogenies. GenBank accession numbers are provided. Scale bar indicates estimated evolutionary distance.

The *glt*A gene sequence (456-bp) obtained from all 20 samples (GenBank accession no. MF693145) showed 100% similarity to the corresponding gene of *R. japonica* strain YH (GenBank accession no. AP017602). The *htr*A gene sequence (394-bp) obtained from 18 samples (GenBank accession no. MF693146) was identical to that of *R. japonica* YH, and the sequence in the remaining 2 samples (GenBank accession no. MF693147) differed by only 3 bp. The *omp*A gene sequence (311-bp) found in 16 samples (GenBank accession no. MF693148) was identical to that of *R. japonica* YH, and the remaining 4 samples each differed by just 1 bp (GenBank accession nos. MF693149–52) (Figure 1).

We also assessed other tickborne agents, including *Anaplasma phygocytophilum*, *Anaplasma capra*, *Ehrlichia chaffeensis*, *Borrelia burgdorferi* sensu lato, *Babesia microti*, and severe fever with thrombocytopenia syndrome virus, by PCR or reverse transcription PCR. Six of 20 patients were co-infected with severe fever with thrombocytopenia syndrome virus, so we excluded them from clinical analysis.

We tested the acute serum samples collected <7 days after illness onset and the convalescence serum samples collected >14 days after illness onset from the 14 patients with *R. japonica* monoinfection in parallel using an indirect immunofluorescence assay for *Rickettsia heilongjiangensis* IgG. We considered an IgG titer >1:64 a positive reaction. Using this definition, we found that 6 patients had a seroconversion, and the other 8 had a 4-fold increased IgG titer; thus, all patients had an acute infection with SFG rickettsiae.

All 14 patients had a history of field activity within the previous month, and 5 had a history of tick bite. Median patient age was 61.5 (range 44–77) years; 9 were men. The median time from tick bite to onset of illness was 4 (range 3–8) days and from onset of illness to physician visit 5 (range 2–7) days. The median duration of hospitalization was 6 (range 4–10) days.

**Figure 2:**
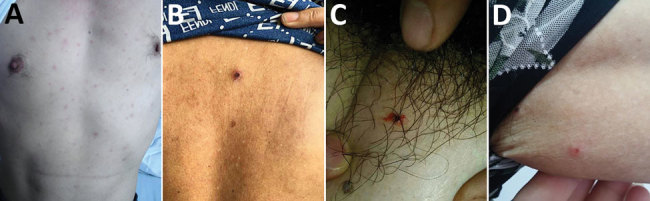
Lesions on patients with *Rickettsia japonica* infection, Xinyang, China, March 2014–June 2017. A) Rash on ventrum; B) rash and eschar on back; C) eschar on femoribus internus; D) tick bite site on left armpit.

Fever and asthenia were reported by all 14 patients. Other nonspecific symptoms included myalgia (10/14), lymphadenopathy (4/14), headache (3/14), dizziness (3/14), and chills (2/14). Gastrointestinal manifestations included anorexia (9/14), nausea (7/14), vomiting (3/14), and diarrhea (1/14). Three patients had cough and pneumonia, and we observed rash in 10 patients (Figure 2). The median time from onset of illness to rash was 4 (range 3–5) days, and the median duration of rash was 5.5 (range 4–8) days. Only 3 patients had an eschar. Other signs included splenomegaly (2/14) and facial edema (1/14).

Urinalysis on admission revealed microscopic hematuria in 2 patients and a slight or moderate proteinuria in 8 patients. The most common findings on laboratory tests were thrombocytopenia, elevated hepatic aminotransferase concentrations, elevated serum lactate dehydrogenase, and hypoalbuminemia, followed by hyponatremia, anemia, hyperbilirubinemia, hypopotassemia, leukopenia, and elevated serum creatine kinase (Table). Mild multiple organ dysfunction developed in 3 patients.

**Table:** Laboratory test results of samples from 14 patients with *Rickettsia japonica* infection at different time points, China, 2014–2017

Result	No. patients		
	At admission	During hospitalization	At discharge from hospital
Anemia, <3.5 × 10^12^ erythrocytes/L	0	5	3
Leukopenia, <4.0 × 10^9^ leukocytes/L	3	3	0
Thrombocytopenia, <150 × 10^9^ platelets/L	9	11	0
Hyperbilirubinemia, albumin >17.1 μmol/L	4	5	2
Hypoalbuminemia, albumin <35.0 g/L	3	9	7
Hyponatremia, sodium <135.0 mmol/L	4	6	2
Hypopotassemia, potassium <3.5 mmol/L	2	4	2
Increased ALT level, >0.67 μkat/L	8	10	5
Increased AST level, >0.67 μkat/L	10	12	5
Increased LDH level, >4.1 μkat/L	10	11	7
Increased CK level, >4.1 μkat/L	4	3	0

*ALT, alanine aminotransferase; AST, aspartate aminotransferase; CK, creatine kinase; LDH, lactate dehydrogenase.

Because of the appearance of typical rash, we suspected SFG rickettsioses in 10 patients and treated them with doxycycline (100 mg 2×/d) until their fevers dissipated and clinical disease improved (median 4 [range 3–5] days). The other 4 patients were treated with the antimicrobial drugs cefminox or cefoperazone for a median of 6 (range 5–7) days. By the time patients were discharged from the hospital, their leukocyte and platelet counts had returned to reference range levels, but half of the patients still had laboratory test values outside of their respective reference ranges (Table). No patients reported clinically significant sequelae at their 2-week follow-up appointment.

## Conclusions

The tick vectors of *R. japonica* (i.e., *H. flava*, *H. hystrici*s, *H. cornigera*, *H. longicornis*, *I. ovatus*) are widely distributed throughout China (*15*), providing *R. japonica* ample opportunity to infect humans. By applying molecular screening techniques to simultaneously amplify 3 genes, we identified *R. japonica* infection in 14 patients.

The clinical signs and symptoms of *R. japonica* infection in our patient cohort differed from those reported in patients in Japan (*3*,*4*). We frequently observed fever, asthenia, and rash but not chills and headache. We also saw fewer eschars in our patient cohort, potentially because they were underreported during clinical examination; eschars are not always easy to identify. In addition, the patients we identified usually had myalgia and gastrointestinal symptoms.

These clinical findings expand the available knowledge of the disease spectrum of *R. japonica* infection. Compared with endemic rickettsiosis caused by *Candidatus* Rickettsia tarasevichiae infection (reference *16* in Appendix), rash is commonly seen, and hemorrhagic and neurologic signs and symptoms are rarely seen in patients with *R. japonica* infection. These distinctive features could be used to make a differential diagnosis.

We frequently observed in our patient cohort thrombocytopenia, hypoalbuminemia, elevated hepatic enzyme activity, and elevated lactate dehydrogenase levels, findings resembling those of patients with common SFG rickettsioses (reference *17* in Appendix). In general, disease is mild or moderate, and no deaths have been recorded, although mild multiple organ dysfunction developed in several patients.

In conclusion, we identified *R. japonica* as an emerging tickborne pathogen in China. Physicians in areas where *H. longicornis* and other competent vectors for *R. japonica* are endemic should be aware of the risk for infection in humans and prescribe doxycycline to patients in cases of ineffective therapy with other antimicrobial drugs. Surveillance needs to be extended to improve our understanding of the health burden of JSF.

Appendix Additional information for *Rickettsia japonica* infection in humans, Xinyang, China, March 2014–June 2017.
