# Supplementary material for: Rickettsia japonica Infections in Humans, Xinyang, China, 2014–2017
Source: Emerg Infect Dis. 2019 Sep;25(9):1719–22. doi: 10.3201/eid2509.171421 (PMC6711240; doi:10.3201/eid2509.171421)
Supplement: Appendix — Additional information for Rickettsia japonica infection in humans, Xinyang, China, March 2014–June 2017. [file 17-1421-Techapp-s1.pdf]

Article DOI: https://doi.org/10.3201/eid2509.171421

Rickettsia japonica Infections in Humans, China, 2014–2017

Appendix

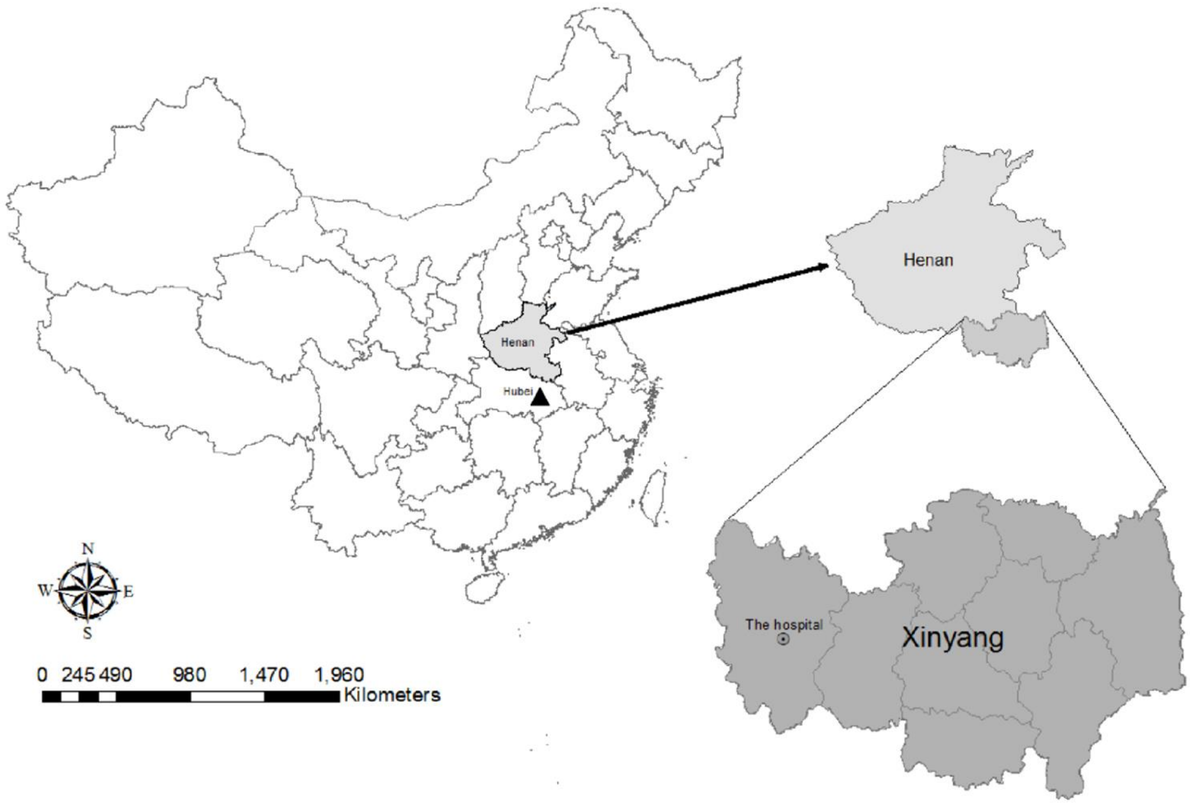

Appendix Figure. Location of Xinyang, Henan Province, China, where the patients with *Rickettsia japonica* infection were detected. The triangle indicates the location of Wuhan, Hubei Province, China, where the ticks of *R. japonica* infection were detected in a previous study (reference *10* in main text).

Appendix References

16. Liu W, Li H, Lu QB, Cui N, Yang ZD, Hu JG, et al. *Candidatus* Rickettsia tarasevichiae infection in eastern central China: a case series. Ann Intern Med. 2016;164:641–8. PubMed https://doi.org/10.7326/M15-2572

17. Parola P, Paddock CD, Socolovschi C, Labruna MB, Mediannikov O, Kernif T, et al. Update on tick-borne rickettsioses around the world: a geographic approach. Clin Microbiol Rev. 2013;26:657–702. PubMed https://doi.org/10.1128/CMR.00032-13
